# Supplementary material for: The Longitudinal Dividing Bacterium Candidatus Thiosymbion Oneisti Has a Natural Temperature-Sensitive FtsZ Protein with Low GTPase Activity
Source: Int J Mol Sci. 2022 Mar 10;23(6):3016. doi: 10.3390/ijms23063016 (PMC8953583; doi:10.3390/ijms23063016)
Supplement: Supplementary file 1 [file ijms-23-03016-s001.zip › ijms-1590011-supplementary.pdf]

International Journal of *Molecular Sciences*

MDPI

Article

The longitudinal dividing bacterium *Candidatus* Thiosymbion oneisti has a natural temperature sensitive FtsZ protein with low GTPase activity

Jinglan Wang [1], Silvia Bulgheresi[2], Tanneke den Blaauwen [1,*]

1 Bacterial Cell Biology and Physiology, Swammerdam Institute for Life Science, University of Amsterdam, 1098 XH Amsterdam, The Netherlands; j.wang3@uva.nl

2 Environmental Cell Biology, University of Vienna, Althanstrasse 14 (UZA I), Vienna 1090, Austria; bulghes3@univie.ac.at

* Correspondence: t.denblaauwen@uva.nl; Tel.: +31-20-525-3852

Abstract: FtsZ, the bacterial tubulin-homolog, plays a central role in cell division and polymerizes into a ring-like structure at midcell to coordinate other cell division proteins. The rod-shaped gamma-proteobacterium *Candidatus* Thiosymbion oneisti has a medial discontinuous ellipsoidal "Z-ring". *Ca.* T. oneisti FtsZ shows temperature-sensitive characteristics when it is expressed in *Escherichia coli*, where it localizes at midcell. Overexpression of *Ca.* T. oneisti FtsZ interferes with cell division and results in filamentous cells. Besides, it forms ring- and barrel-like structures independently of *E. coli* FtsZ, which suggests that the difference in shape and size of the *Ca.* T. oneisti FtsZ ring is likely the result of its interaction with Z-ring organizing proteins. Similar to some temperature-sensitive alleles of *E. coli* FtsZ, *Ca.* T. oneisti FtsZ has a weak GTPase and does not polymerize *in vitro*. The temperature sensitivity of *Candidatus* Thiosymbion oneisti FtsZ is likely an adaptation to the preferred temperature of less than 30 °C of its host the nematode *Laxus oneistus.*

Keywords: Bacterial cytoskeleton; cell division; FtsZ assembly; temperature sensitive

Supplementary materials

Materials and Methods

Plasmid construction

The *ftsZ*^{TO} gene and *mreB*^{TO} gene were amplified from *Ca*. T. oneisti DNA by PCR reaction. The genome sequence draft is available at http://rast.nmpdr.org/rast.cgi. Methanol fixed nematodes were rehydrated in Phosphate Buffered Saline (PBS: pH 7.4; 137 mM NaCl, 2.7 mM KCl, 10 mM Na_2HPO_4, 1.8 mM NaH_2PO_4), and symbiont bacteria were detached in a sonication bath for 1 min sonication. One microliter bacterial suspension was used as template for 25 µL PCR reaction. Primers used for plasmid construction are listed in Table S1.

Plasmid pJW11 was created by first amplifying the *ftsZ*^{TO} gene from *Ca*. T. oneisti DNA using primers priJW82 and priJW83. PCR condition was as follows: 98 °C for 10 min, followed by 30 cycles at 98 °C for 30 s, 57 °C for 30 s, 72 °C for 90 s, followed by a final elongation step at 72 °C for 10 min. Next, the backbone vector pSAV057 [2] was amplified by priJW14 and priJW20. PCR condition was as follows: 98 °C for 10 min, followed by 30 cycles at 98 °C for 30 s, 57 °C for 30 s, 72 °C for 5 min, followed by a final elongation step at 72 °C for 10 min. pJW11 was constructed by Gibson assembly [1].

Plasmid pJW12 was created by first amplifying the *ftsZ*^{EC} gene from *E. coli* strain LMC500 using primers priJW88 and priJW89. PCR condition was as follows: 98 °C for 10 min, followed by 30 cycles at 98 °C for 30 s, 53 °C for 30 s, 72 °C for 90 s, followed by a final elongation step at 72 °C for 10 min. Next, the backbone vector pSAV057 [2] was amplified by priJW14 and priJW20 as described before. pJW12 was constructed by Gibson assembly.

Plasmid pJW13 was created by first amplifying the *ftsZ*^{TO} gene from *Ca*. T. oneisti DNA using primer priJW119 and priJW120. PCR condition was same as priJW82/priJW83. Next, the amplified *ftsZ*^{TO} gene product and pSAV047-mCherry vector [2] were digested using *Eco*RI and *Hind*III and subsequently ligated to create pJW13.

Plasmid pJW14 was created by first amplifying the *ftsZ*^{EC} gene from *E. coli* strain LMC500 using priJW119/priJW120. PCR condition was same as priJW88/priJW89. Next, the backbone vector pSAV047-mCherry vector [1] was amplified by priJW121/priJW122. PCR condition was as follows: 98 °C for 10 min, followed by 30 cycles at 98 °C for 30 s, 56 °C for 30 s, 72 °C for 5 min, followed by a final elongation step at 72 °C for 10 min. pJW14 was constructed by Gibson assembly.

Plasmid pJW15 was created by first amplifying the *ftsZ*^{TO} gene from plasmid pJW11 using priJW98/priJW99 and the *mCherry-ftsZ*^{TO} from plasmid JW13 using priJW95/priJW96. Next, the backbone vector pBAD was amplified by priJW94 and priJW97. PCR condition was as follows: 98 °C for 10 min, followed by 30 cycles at 98 °C for 30 s, 57 °C for 30 s, 72 °C for 5 min, followed by a final elongation step at 72 °C for 10 min. Three fragments of pJW15 were assembled by Gibson assembly.

Plasmid pJW16 was created by first amplifying the *mCherry-ftsZ*^{TO} from plasmid JW13 using. priJW95 and priJW96. Next, the backbone vector pBAD was amplified by priJW93 and priJW97 as described before. pJW16 was constructed by Gibson assembly.

Plasmid pJW17 was created by first amplifying the *ftsZ*^{TO} gene from *Ca*. T. oneisti DNA using primers priJW and priJW. PCR condition was same as priJW82/priJW83. Next, the amplified *ftsZ*^{TO} gene product and pET11b vector were digested using *Nde*I and *Bam*HI and subsequently ligated to create pJW17.

Plasmid pJW18 was created by amplifying the *mreB*^{TO} gene from *Ca*. T. oneisti DNA using primers priJW1 and priJW2. PCR condition was as follows: 98 °C for 10 min, followed by 30 cycles at 98 °C for 30 s, 58 °C for 30 s, 72 °C for 90 s, followed by a final elongation step at 72 °C for 10 min. Next, the backbone vector pTB146 (PT7-lac::His-sumo, a gift from Martin Loose, Institute of Science and Technology Austria) was amplified by priJW3 and priJW4. PCR condition was as follows: 98 °C for 10 min, followed by 30 cycles at 98 °C for 30 s, 57 °C for 30 s, 72 °C for 4 min, followed by a final elongation step at 72 °C for 10 min. pJW18 was constructed by Gibson assembly.

Plasmid pJW19 was created by site-directed mutagenesis from pJW18 using primers priJW78 and PriJW79.

Plasmid pJW20 was created by site-directed mutagenesis from pJW17 using primers priJW117 and priJW118.

Plasmid pJW21 was created by site-directed mutagenesis from pJW17 using primers priJW115 and priJW116.

Table S1. Primers used in this study.

Primer	Sequence 5′-3′	use
priJW82	TTTCACACAGGAAACAGACCATGTTTGAACTAGTGGATACCA	pJW11 and pJW13 construction
priJW83	GTTCGGGCCCAAGCTCATTATCAGTCCGCCTGATGGCGCA	pJW11 and pJW13 construction
priJW14	GGTCTGTTTCCTGTGTGAAATTG	pJW11 and JW12 construction
priJW20	TAATGAGCTTGGGCCCGAACAA	pJW11 and JW12 construction
priJW88	TCACACAGGAAACAGACCATGTTTGAACCAATGGAACT	pJW12 construction
priJW89	GTTCGGGCCCAAGCTCATTATTAATCAGCTTGCTTACGC	pJW12 construction
priJW119	GGGGGGGAATTCATGTTTGAACTAGTGGATACC	pJW13 construction
priJW120	GGGAAGCTTTCAGTCCGCCTGATGG	pJW13 construction
priJW119	CCGCCAAAACAGCCAAGCTTTAATCAGCTTGCTTACGC	pJW14 construction
priJW120	GACGAGCTGTACAAGGAATTCATGTTTGAACCAATGGAACT	pJW14 construction
priJW121	CATGAATTCCTTGTACAGCTCGTC	pJW14 construction
priJW122	AAGCTTGGCTGTTTTGGCGG	pJW14 construction
priJW94	TGCGCCATCAGGCGGACTGAGATCCGAGCTCGAGATCTGC	pJW15 construction
priJW95	ATGGTGAGCAAGGGCGAGGA	pJW15 and JW16 construction
priJW96	TCAGTCCGCCTGATGGCGCA	pJW15 and JW16 construction
priJW97	GGTTAATTCCTCCTGTTAGCC	pJW15 and JW16 construction
priJW98	GCTAACAGGAGGAATTAACCATGTTTGAACTAGTGGA-TACCA	pJW15 construction
priJW99	TCCTCGCCCTTGCTCACCATTCAGTCCGCCTGATGGCGCA	pJW15 construction
priJW93	TCCTCGCCCTTGCTCACCATGGTTAATTCCTCCTGTT	pJW16 construction
priJW123	GATATACATATGTTTGAACTAGTGGATACC	pJW17 construction
priJW124	AGCCGGATCCTCAGTCCGCCTGATGG	pJW17 construction
priJW1	TCACAGAGAACAGATTGGTGGGATGTTTCTCCGACGCATTCG	pJW18 construction
priJW2	CTTCCTGCAGTCACCCGGGCCTCCGTCATAGCCAACAGGT	pJW18 construction
priJW3	GCCCGGGTGACTGCAGGAAG	pJW18 construction
priJW4	CCCACCAATCTGTTCTCTGTGA	pJW18 construction
priJW78	CTCGGCGACAGCGGCACCCAGCTCCGGA	pJW19 construction
priJW79	GAGCTGGGTGCCGCTGTCGCCGAGCG	pJW19 construction
priJW117	CCTGGCCTCATGAATGTGGACTTTGCGGATGTCAAGACCGT	pJW20 construction
priJW118	TCCACATTCATGAGGCCAGGACGGGTGATGAGCTCG	pJW20 construction
priJW115	AGCTTGTCCGAGGGTGAGTTCGACGAGGTGGGCAATAC-GGTG	pJW21 construction
priJW116	AACTCACCCTCGGACAAGCTCATGCCGGCGGTGATGTTGAC	pJW21 construction

Table S2. GTPase activity (μM Pi/μM FtsZ/min) of FtsZ^{TO}.

GTP concentration	1 mM	2 mM	3 mM	4 mM	5 mM
**FtsZ^{TO} batch 1 (in MES buffer)**	1,602	1,785	2,052	2,135	2,138
**FtsZ^{TO} batch 2 (in MES buffer)**	1,595	1,601	1,920	1,812	2,094
**FtsZ^{TO} batch 3 (in MES buffer)**	1,796	1,910	2,247	2,623	2,536
**I207M batch 1 (in MES buffer)**	0,325	N/A	N/A	N/A	N/A
**I207M batch 2 (in MES buffer)**	0,303	N/A	N/A	N/A	N/A
**I207M batch 3 (in MES buffer)**	0,338	N/A	N/A	N/A	N/A
**I273M batch 1 (in MES buffer)**	0,824	N/A	N/A	N/A	N/A
**I273M batch 2 (in MES buffer)**	0,794	N/A	N/A	N/A	N/A
**I273M batch 3 (in MES buffer)**	0,962	N/A	N/A	N/A	N/A
**FtsZ^{EC} batch 1 (in MES buffer)**	9,378	N/A	N/A	N/A	N/A
**FtsZ^{EC} batch 2 (in MES buffer)**	9,017	N/A	N/A	N/A	N/A
**FtsZ^{EC} batch 3 (in MES buffer)**	10,304	N/A	N/A	N/A	N/A
**FtsZ^{TO} batch 1 (in HEPES buffer)**	1,521	N/A	N/A	N/A	N/A
**FtsZ^{TO} batch 2 (in HEPES buffer)**	1,067	N/A	N/A	N/A	N/A
**FtsZ^{TO} batch 3 (in HEPES buffer)**	1,619	N/A	N/A	N/A	N/A
**I207M batch 1 (in HEPES buffer)**	0,122	N/A	N/A	N/A	N/A
**I207M batch 2 (in HEPES buffer)**	0,115	N/A	N/A	N/A	N/A
**I207M batch 3 (in HEPES buffer)**	0,153	N/A	N/A	N/A	N/A
**I273M batch 1 (in HEPES buffer)**	0,543	N/A	N/A	N/A	N/A

**I273M batch 2 (in HEPES buffer)**	0,527	N/A	N/A	N/A	N/A
**I273M batch 3 (in HEPES buffer)**	0,605	N/A	N/A	N/A	N/A

Table S3. Buffers for light scattering of FtsZ^{TO}.

50 µM HEPES, 50 mM KCl, 5 mM $MgCl_2$, 1mM GTP, pH7.5
50 µM HEPES, 50 mM KCl, 5 mM $MgCl_2$, 10 mM GTP, pH7.5
50 µM HEPES, 50 mM NaCl, 5 mM $MgCl_2$, 1mM GTP, pH7.5
50 µM HEPES, 50 mM KCl, 10 mM $MgCl_2$, 1mM GTP, pH7.5
50 µM HEPES, 50 mM KCl, 5 mM $MgCl_2$, 5 mM $CaCl_2$, 1mM GTP, pH7.5
50 µM HEPES, 50 mM KCl, 5 mM $MgCl_2$, 5 mM EDTA, 1mM GTP, pH7.5
50 µM HEPES, 50 mM KCl, 5 mM Mg-acetate, 1mM GTP, pH7.5
50 µM MES, 50 mM KCl, 5 mM $MgCl_2$, 1mM GTP, pH6.5
50 µM MES, 50 mM KCl, 5 mM $MgCl_2$, 10mM GTP, pH6.5
50 µM MES, 50 mM NaCl, 5 mM $MgCl_2$, 1mM GTP, pH6.5
50 µM MES, 50 mM KCl, 10 mM $MgCl_2$, 1mM GTP, pH6.5
50 µM MES, 50 mM KCl, 5 mM $MgCl_2$, 5 mM $CaCl_2$, 1mM GTP, pH6.5
50 µM MES, 50 mM KCl, 5 mM $MgCl_2$, 5 mM EDTA, 1mM GTP, pH6.5
50 µM Tris-HCl, 50 mM KCl, 5 mM $MgCl_2$, 1mM GTP, pH7.5
50 µM Tris-HCl, 50 mM NaCl, 5 mM $MgCl_2$, 1mM GTP, pH7.5

Supplementary figures

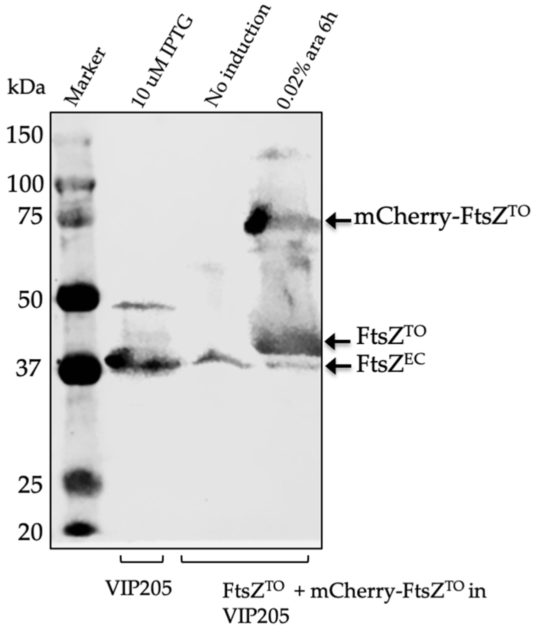

Figure S1. $FtsZ^{TO/EC}$ protein expression level. Western blot image shows the expression of $FtsZ^{TO}$, mCherry-$FtsZ^{TO}$, $FtsZ^{EC}$ in VIP205 strain cultured in TY medium at 28 °C with or without addition of 10 μM IPTG or 0.02% arabinose for 6 h.

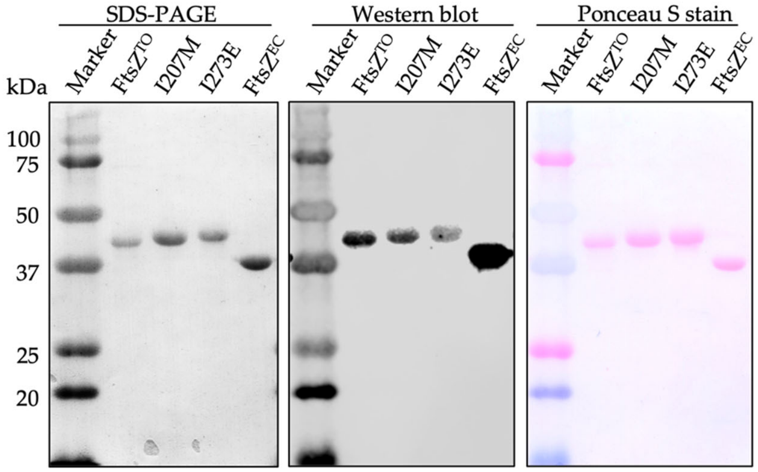

Figure S2. Purification of $FtsZ^{EC}$, $FtsZ^{TO}$ and $FtsZ^{TO}$ variants. SDS-PAGE (left) detected by Coomassie Blue staining, Western blot (middle) and corresponding ponceau S stain (right) of purified $FtsZ^{TO}$, $FtsZ^{TO}$ I207M, $FtsZ^{TO}$ I273E and $FtsZ^{EC}$.

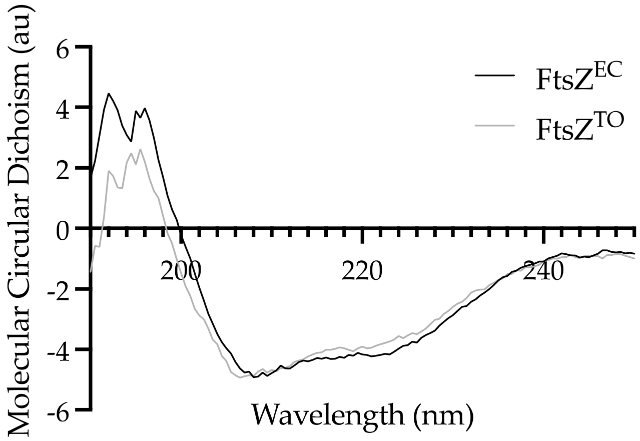

Figure S3. FtsZ^{EC} and FtsZ^{TO} show similar far UV circular dichroism spectra.

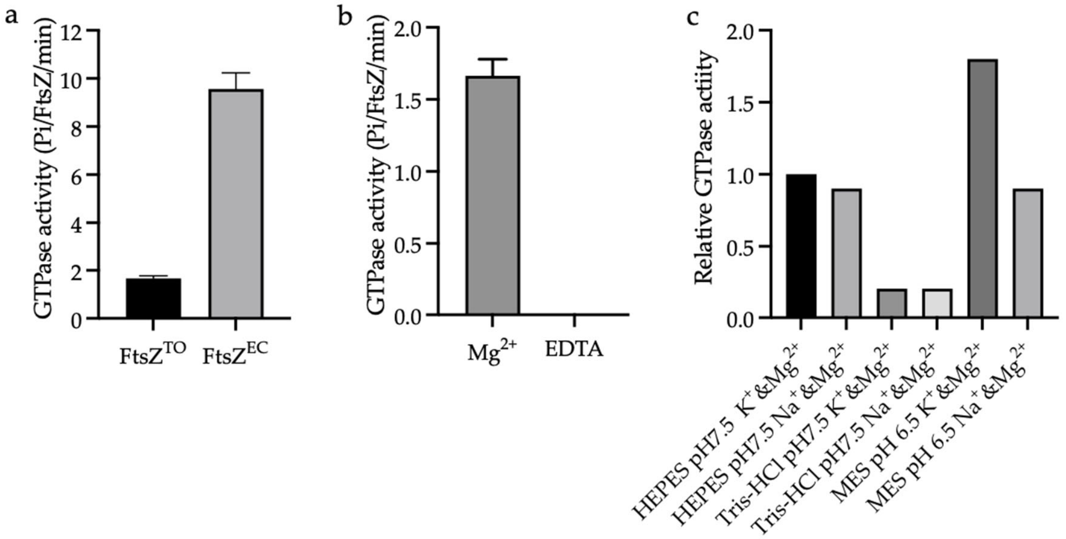

Figure S4. GTPase activity of FtsZ$^{TO/EC}$. (**a**) Comparison of GTPase activities of FtsZ^{TO} and FtsZ^{EC}. FtsZ$^{EC/TO}$ (5 μM) were carried out in MES buffer (50 mM MES, 50 mM KCl, 5 mM $MgCl_2$, pH 6.5), 1 mM GTP, at 28 °C. Error bars are the standard error of the mean, n = 3 independent experiments. (**b**) GTPase activities of FtsZ^{TO} (5 μM) were carried out in MES buffer (50 mM MES, 50 mM KCl, pH 6.5) with 5 mM $MgCl_2$ or with 5mM EDTA, 1 mM GTP, at 28 °C. Error bars are the standard error of the mean, n = 3 independent experiments. (**c**) Relative GTPase activities of FtsZ^{TO} in 50 mM HEPES buffer, 50 mM MES buffer or 50 mM Tris-HCl buffer with 50 mM KCl or NaCl, 5 mM $MgCl_2$, 1 mM GTP, at 28 °C. The GTPase activity in 50 mM HEPES buffer, 50 mM KCl, 5 mM $MgCl_2$ was set as control.

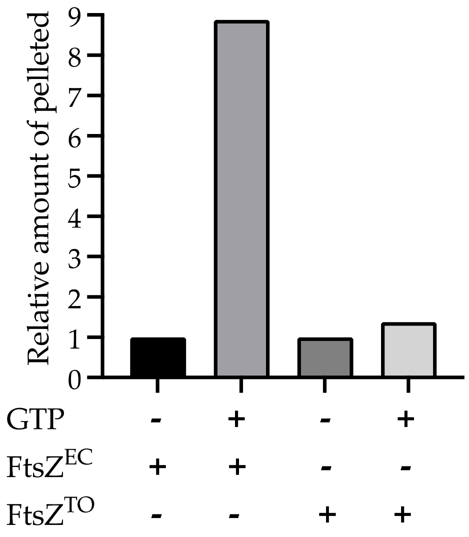

Figure S5. Sedimentation assay of $FtsZ^{TO}$ and $FtsZ^{EC}$. $FtsZ^{EC/TO}$ (9 µM) was polymerized with GTP (1 mM) in MES buffer (50 mM MES, KCl 50 mM, $MgCl_2$ 10mM, $CaCl_2$ 10mM, pH 6.5) for 10 min at 28°C and spun down at 240,000 x g for 20 min at 4°C. The amount of $FtsZ^{EC/TO}$ pelleted in absence of GTP was set as control.

```
FtsZTO    1 MFELVD-TNGQDAVIKVIGVGGGGGNAVNQMVESSIEGVDFICANTDSQA    49
            |||.:: ||  |||||||||||||||||..||...||||:|...|||:||
FtsZEC    1 MFEPMELTN--DAVIKVIGVGGGGGNAVEHMVRERIEGVEFFAVNTDAQA    48

FtsZTO   50 LKSSNVKTILQLGADITKGLGAGADPKVGKEAALEDKERIHDALEGADMV    99
            |:.:.|...:|:|:.|||||||||:|:||:.||.||::.:..||||||||
FtsZEC   49 LRKTAVGQTIQIGSGITKGLGAGANPEVGRNAADEDRDALRAALEGADMV    98

FtsZTO  100 FITAGMGGGTGTGAAPVVAQVARELGILTVAVVTKPFAFEGAKRMKVAVE    149
            ||.||||||||||||||||:||::|||||||||||||.|||.|||..|.:
FtsZEC   99 FIAAGMGGGTGTGAAPVVAEVAKDLGILTVAVVTKPFNFEGKKRMAFAEQ    148
          tubulin signature motif
FtsZTO  150 GISELASHVDSLITIPNEKLLAVLGKEMSLLNSFKAANDVLLNATQGIAE    199
            ||:||:.||||||||||:|||.|||:.:|||::|.||||||..|.|||||
FtsZEC  149 GITELSKHVDSLITIPNDKLLKVLGRGISLLDAFGAANDVLKGAVQGIAE    198
                                                    H7
FtsZTO  200 LITRPGLINVDFADVKTVMAEMGQAMMGTGIASGEQRAREAAEAAINSPL    249
            |||||||:|||||||:|||:|||.||||:|:||||.||.||||.||:|||
FtsZEC  199 LITRPGLMNVDFADVRTVMSEMGYAMMGSGVASGEDRAEEAAEMAISSPL    248
                    T7
FtsZTO  250 LEDIDLAGAKGILVNITAGMSLSIGEFDEVGNTVRDFADDDAIVVVGTVI    299
            ||||||:||:|:|||||||..|.:.||:.||||:|.||.|:|.||:||.:
FtsZEC  249 LEDIDLSGARGVLVNITAGFDLRLDEFETVGNTIRAFASDNATVVIGTSL    298
                                     H10
FtsZTO  300 DPDLSEELRVTVVATGLGEGRRAQLSKPAMGESPIHLVPRGGTGSVTPDY    349
            |||:::||||||||||:|..:|.:::          ||..
FtsZEC  299 DPDMNDELRVTVVATGIGMDKRPEIT----------LVTN----------    328

FtsZTO  350 RDMERPAVYRKGYQGVAAAA-----VAQAHPDAVEET--DLDYLDIPAFL    392
            :.:::|.:.|....|:|...     ||:...|...:|  :.|||||||||
FtsZEC  329 KQVQQPVMDRYQQHGMAPLTQEQKPVAKVVNDNAPQTAKEPDYLDIPAFL    378
                         CTL sequence              CTD sequence
FtsZTO  393 RHQAD    397
            |.|||
FtsZEC  379 RKQAD    383
```

Figure S6. Alignment of FtsZ^{TO} and FtsZ^{EC}. Residues contacting with GTP or GDP in the crystal structure are shown in red. Tubulin motif in blue, C-terminal linker (CTL) and C-terminal domain (CTD) are labelled. Sequence identity: 61.2% (248/405); sequence similarity: 75.6% (306/405); gaps: 7.4% (30/405).

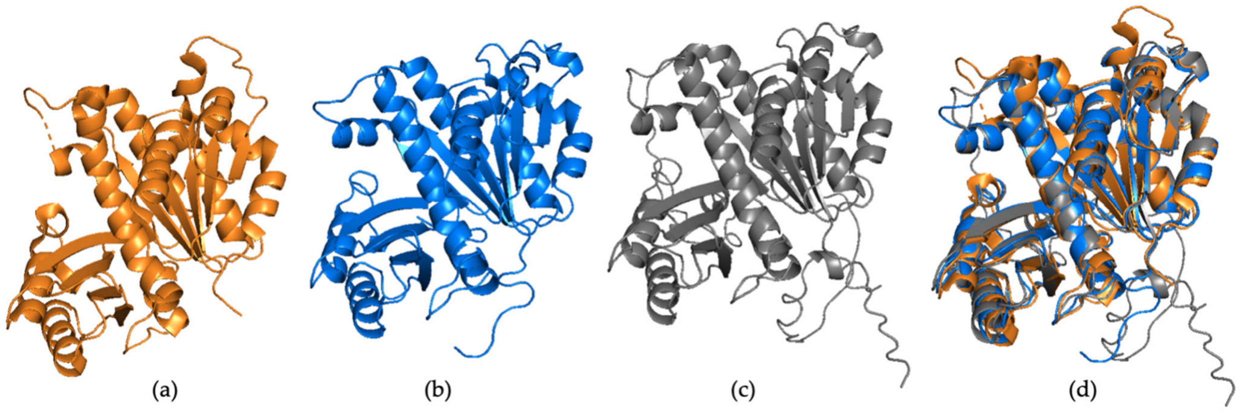

Figure S7. Predicted structure model of FtsZ^{TO}. (**a**) FtsZ^{EC} crystal structure (PDB entry 6UNX). (**b**) FtsZ^{TO} structure model from Phyton2. (**c**) FtsZ^{TO} structure model from Alphafold2. (**d**) Structure alignment of two FtsZ^{TO} models and FtsZ^{EC}. Structures are colored same as in (a), (b), and (c).

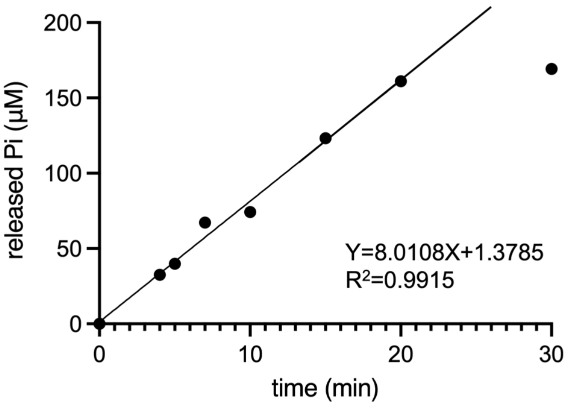

Figure S8. Example of GTP hydrolysis rates calculation. GTP (1 mM) was added into FtsZ^{TO} (5 μM) in MES buffer (50 mM MES, 50 mM KCl, 5 mM $MgCl_2$, pH 6.5) at 28°C to calculate the GTPase activity of FtsZ^{TO}. At 30 min, all GTP had been hydrolyzed and points from 0 min to 20 min were used to calculate the slope of the linear increase, which is the GTP hydrolysis rate (Here is 8,0108 μM Pi/min) of 5 μM FtsZ^{TO} at the corresponding condition. The slope is divided by the FtsZ concentration (5 μM) to obtain the GTPase activity (1.602 μM Pi/μM FtsZ/min) of FtsZ^{TO}. All GTPase activity data of FtsZ^{TO} are listed in Table S2.

References

1. Gibson, D. G.; Young, L.; Chuang, R.; Venter, J. C.; Hutchison, C. A.; Smith, H. O. Enzymatic assembly of DNA molecules up to several hundred kilobases. *Nat. Methods*. **2009**, *6*, 343-345.

2. Meiresonne, N. Y.; Consoli, E.; Mertens, L. M. Y.; Chertkova, A. O.; Goedhart, J.; Blaauwen, den, T. Superfolder mTurquoise2ox optimized for the bacterial periplasm allows high efficiency *in vivo* FRET of cell division antibiotic targets. *Mol. Microbiol.* **2019**, *111*, 1025–1038.
